# Bipolar disorders and creativity: the roles of ambition, effort-based decision-making, and exploration

**DOI:** 10.3389/fpsyt.2025.1505314

**Published:** 2025-04-29

**Authors:** Samson Tse, Sheri L. Johnson, Chong Ho Yu, Winnie Yuen, Iris Lo, Luke Clark, Erin E. Michalak, Manon Ironside, Kiana Modavi

**Affiliations:** ^1^ Department of Social Work and Social Administration, The University of Hong Kong, Pokfulam, Hong Kong SAR, China; ^2^ Department of Psychology, University of California, Berkeley, Berkeley, CA, United States; ^3^ Department of Mathematics, Hawaii Pacific University, Honolulu, HI, United States; ^4^ Department of Counselling and Psychology, Hong Kong Shue Yan University, North Point, Hong Kong SAR, China; ^5^ Centre for Gambling Research at UBC, Department of Psychology, Vancouver, BC, Canada; ^6^ Djavad Mowafaghian Centre for Brain Health, Vancouver, BC, Canada

**Keywords:** exploration versus exploitation, recovery, strength based approach, creative accomplishments, Chinese community

## Abstract

**Introduction:**

Epidemiological research has shown those with bipolar disorders (BD) are more likely to work in creative professions. The current work is the first to examine ambition, exploration versus exploitation ratio, and insensitivity to effort/rewards among individuals with and without BD in an Asian cultural context.

**Methods:**

Writers and visual artists from Hong Kong who were diagnosed with BD completed a questionnaire to assess lifetime creative accomplishments, a self-rated measure of ambition, and two laboratory-based tasks: the observe-or-bet task to detect exploration versus exploitation tendencies, and an effort discounting task to measure sensitivity to effort required and reward level.

**Results:**

The sample included 44 participants diagnosed with BD and 69 control participants, with 87 (77%) being female and an average age of 35.1 years (range: 18 to 65). Bayesian analyses found no group differences in creativity or related mechanisms between BD and control participants. However, decision tree algorithms revealed multivariate contributors to creative accomplishments. Replicating prior work, high ambition was key, with the most productive also willing to persevere despite high effort. Among lower-ambition individuals, control participants who engaged in balancing exploration versus exploitation had greater accomplishments. Importantly, there was no evidence that the effects of ambition or effort-based decision-making on creativity differed based on BD diagnosis. Bipolar group had lower socioeconomic status potentially impacting their self-rated creativity scores and creative potential. However, these findings remain tentative and await further investigation due to limited sample size.

**Discussion:**

The findings suggest the mechanisms underlying creativity may not inherently differ for those with BD compared to controls. Ambition, especially when combined with effort and willingness, drives creative accomplishments. Strategic use of exploration versus exploitation was associated with greater creativity among less ambitious individuals without BD. A nuanced, multivariate approach is needed to understand the bipolar-creativity relationship across cultures. Study limitations included small sample size and over-representation of female participants.

## Introduction

Bipolar disorders (BD) affects 0.7% of the adult population globally (1). In Hong Kong, the 12-month prevalence rates of bipolar I (BD-I) and bipolar II disorder (BD-II) were 1.4% and 0.5%, respectively, in 2009 (2). BD is characterized by acute episodes of depressed, manic, and mixed mood states, causing considerable impairments for those affected. BD has been tied to high rates of unemployment, disability, and suicidality.

Creativity refers to a person’s multimodal capacity to produce thought, behaviors, or products that are novel and original, as well as functional. Creativity can be expressed in laboratory tasks, hobbies, or occupations in the real world and is known to be related to specific personality traits (e.g., openness to experience and extraversion; 3).

Non-academic literature, as well as research findings, support the idea that individuals with BD are significantly more likely to engage in creative professions than those without BD (4). Notable examples span famous authors, artists, and actors who showed symptoms of BD (5), including Vincent van Gogh, Edvard Munch, Virginia Woolf, and Francisco de Goya. BD is notably the only psychiatric illness to show such clear links with creative occupations (6).

However, some individuals with BD do poorly in terms of achieving creative or life goals at a broad level (7, 8). In one study, people with BD did not differ from non-BD controls in their mean level of creativity, demonstrating instead greater variability in their degree of creativity (9). It has been argued that greater variability could help explain the presence of those with BD among highly eminent groups.

A core goal is to explain the variability observed in creative accomplishments within the BD group (7). Research suggests several correlates of BD that may enhance creativity. For example, BD is linked to increased activity in the neurobiological reward system (7), resulting in a greater willingness to work hard for less reward (10) and higher ambitions for success (11), both associated with elevated creativity (12, 13). Additionally, BD is related to a greater willingness to explore novel situations for potential rewards (14), which is essential for generating creative solutions (15). Despite these insights, research integrating these factors is scarce. This study aims to fill that gap.

## Present study: bipolar disorders and creativity

Our aim is to examine whether certain psychological aspects of reward pursuit —specifically ambition, willingness to expend effort, and exploration versus exploitation in decision-making— act as behavioral indicators of creativity in BD (16). To assess willingness to expend effort, we employed an Effort Discounting Task. In this task, participants choose between squeezing a handgrip to earn a higher monetary reward (higher effort) or accepting a smaller amount with no effort (lower effort) (17). As effort requirements increase, participants typically shift their choices toward the easier option, demonstrating effort discounting. Reward amounts also differ across trials, to provide an index of sensitivity to monetary reward. We also used the Observe or Bet Task (18) to evaluate the trade-off between exploration and exploitation. In foraging scenarios, reward seeking involves balancing the exploration of options with unknown value to gather information against exploiting options with known value, even when better alternatives may exist (19). We hypothesize that participants with BD will demonstrate greater effort in reward pursuit and make more exploratory decisions compared to exploitative ones.

## Method

Ethical approval was obtained from The University of Hong Kong Human Research Ethics Committee for Non-Clinical Faculties.

### Participants

Adults aged 18 to 70 years with primary BD-I and BD-II and age-matched adults with no current or history of mood or psychotic disorders, as assessed by the Structured Clinical Interview for DSM-5 (SCID-5), were recruited for the present study. Additional inclusion criteria for both groups included being ethnically Chinese and currently participating in at least two hours per week or eight hours per month of creative activities related to writing and/or painting. Exclusion criteria included: the inability to provide written informed consent; the inability to concentrate for more than 15 minutes during the study tasks or measures, and the use of medications or presence of illnesses that could impair cognitive functioning or interfere with task completion, alongside any other indications of impaired mental status; any history of brain injury or neurological illness; the presence of substance use disorder within the past year; participation in electroconvulsive therapy within the past year; or being a danger to the self or others.

Focusing on creativity in writing and visual art is a distinctive feature of this study. In contrast to many studies on creativity in individuals with BD, which tend to use a broad definition, we specifically examine these two domains. However, our intent was not to compare the psychological processes between different participant groups.

### Materials and procedures

Participants completed phone screening for the exclusion criteria and then completed the other measures in an in-person session at the university or another location suitable for private data collection (for details on the measuring instruments, see supplementary information on measuring instruments). Basic sociodemographic information was collected, including age, gender, education level, and occupation.

#### Carson creative achievement questionnaire

Our primary dependent variable was the CAQ, a self-report measure designed to assess lifetime creative accomplishments across 10 domains: visual arts, music, creative writing, dance, drama, architecture, humor, science, invention, and culinary skills (20). Sample statements include: “My work has won an award or prize”, “my work has been printed and sold publicly”, and “my work has been reviewed in national publications.” The CAQ was augmented with a further item “Do people describe you as creative?” and were asked to respond either “yes” or “no” which was reported separately.

#### Willingly approached set of statistically unlikely pursuits: popular fame and financial success subscales

The WASSUP Popular Fame and Financial Success Subscales, designed to assess extremely high life ambitions (Johnson & Carver, 2006), were highly intercorrelated (r = 0.66) (21).

#### Effort discounting task

The Effort Discounting Task is a well-validated procedure designed to assess a person’s willingness to work harder to earn monetary rewards (17). The task was programmed in Python, with keyboard control, and a Biopac hand dynamometer TSD121c device with a DA100C transducer synchronized with the task delivery computer through the parallel port, using a MP160WSW data. Before the task began, participants were asked to squeeze the hand dynamometer as hard as they could for two seconds. Then, they were offered a chance to earn a bonus monetary reward if they could increase their grip pressure in a two-second round. This calibration was used to then index the level of effort, as a percentage of the maximum grip strength. The monetary rewards accumulated as the task progressed were paid as a bonus at completion. Participants were remunerated up to HK$200 (approx. US$25.6) each for their performance in the task.

#### Observe or bet task

This task is designed to assess people’s tendency to make exploratory versus exploitative choices (18). In each block of 50 trials, the participants viewed a sequence of blue or orange lights, with one color biased to appear 75% of the time. In each trial, the participant made a choice between simply observing the light appear (an exploratory decision that did not yield any points) or guessing the color (“bet”, an exploit decision). On bet choices, the participant receives no feedback on the light color, and thus this task can fully separate information-seeking (explore) and reward-maximizing (exploit) choices.

#### Young mania rating scale

The YMRS is an interview-based rating scale designed to evaluate current manic symptom severity (22).

#### Positive and negative affect schedule

The PANAS is a self-report questionnaire with 10 positive and 10 negative affect descriptors regarding mood and mental distress during the past week (23).

#### Brief version of quality of life in BD

The Brief QoL.BD is a well-validated self-report scale designed to capture QoL in individuals with BD across 14 domains (24, 25).

### Data analyses

Bayesian statistics and data science methods were employed for analysis in an attempt to overcome issues that are prevalent in classical statistics, such as overfitting. Specifically, Bayesian t-tests were used to evaluate diagnostic group differences (BD or non-BD) in creativity, ambition, effort-based decision-making, and explore versus exploit decision-making. Bayesian statistical approaches used here differ from traditional frequentist methods, which interpret probability based on long-term relative frequencies and use p-values to indicate the likelihood of observing the test statistic under the null hypothesis. In contrast, Bayesian techniques assess the strength of evidence for hypotheses based on the observed data, without relying on theoretical sampling distributions (26, 27). Bayesian analysis directly compares the evidence for the null and alternative hypotheses using Bayes factors, offering a continuous measure of support rather than a binary decision based on a fixed threshold (28, 29). Further, most data science methods, such as the decision tree, are non-parametric in essence. Thus, there is no need to assume any specific sampling distributions or data structure in the sample. Instead, the conclusion is drawn by detecting the patterns in the data (30). Data visualization, as a major component of data science, also aims to facilitate pattern recognition.

To analyze the factors related to our creativity outcome (CAQ total scores), we used decision tree algorithms. This supervised learning method employs a flowchart-like structure to model both continuous and categorical outcomes, helping identify which independent variables can effectively split the data into subgroups. Unlike ordinary least squares regression, this approach is unaffected by multicollinearity. We conducted the analyses using JASP (31) and JMP Pro (32). This method allowed us to examine how varying levels of ambition, sensitivity to rewards or effort, exploration versus exploitation tendencies, and diagnostic group status relate to creativity. We did not report the decision tree results separately for creative writing and painting for two reasons: smaller sample sizes would limit the robustness of the findings, and many participants engaged in both activities, complicating the meaningful separation of the two forms of creativity.

## Results

The study recruited a total of 113 participants, including 44 individuals with BD and 69 without, all of whom successfully completed the laboratory tasks and surveys.

As shown in **Table 1**, the BD and non-BD groups differed in several ways. A smaller proportion of the BD group completed undergraduate or postgraduate studies than the non-BD group, and. participants with BD were less likely to be in full-time or part-time employment than those without BD as indicated by a large Bayes factor. The BD group exhibited higher mean levels of manic symptoms and negative affect compared to the non-BD group, while also reporting lower quality of life scores (see **Table 1**).

**Table 1:** Descriptive and Bayes factors of sociodemographic factors.

	Bipolar (n = 44)		Non-bipolar (n = 69)		^1^BF_10_
**Age**	Mean = 35.11		Mean = 35.20		0.20
	Std dev = 12.82		Std dev = 13.24		
	n	Column %	n	Column %	
**Gender**					0.31
Female	36	81.82%	51	73.91%	
Male	8	18.18%	18	26.09%	
**Education**					2.14
Primary school	0	0%	0	0%	
Junior high school	2	4.55%	3	4.35%	
High school	9	20.45%	5	7.25%	
College or university	29	65.91%	40	57.97%	
Postgraduate or above	4	9.09%	21	30.43%	
**Occupation**					224.42
Student	5	11.36%	20	28.99%	
PT work	8	18.18%	6	8.70%	
FT work	12	27.27%	30	43.48%	
Self-employed	2	4.55%	7	10.14%	
Not in employment	13	29.55%	3	4.35%	
Retired	4	9.09%	3	4.35%	
**^2^Young Mania Rating Scale (YMRS)**	Mean = 3.39 Std dev = 3.45		Mean = 0.93 Std dev = 2.33		1170.33
**^3^Positive Affect Score**	Mean = 30.34 Std dev = 6.83		Mean = 28.20 Std dev = 6.45		0.942
**Negative Affect Score**	Mean = 24.72 Std dev = 8.60		Mean = 19.80 Std dev = 6.58		167.80
**^4^Brief Quality of Life Scale for Bipolar Disorder (Brief QoL.BD)**	Mean = 41.09 Std dev = 8.16		Mean = 44.12 Std dev = 6.82		2,61

^1^Bayes factor of the independent multinomial.

Null: The row and the column cell counts are independent; alternate: There is substantial dependency (relationship).

< 1: favors the null hypothesis; > 1: favors the alternate hypothesis (underlined); = 1: neutral.

^2^The Cronbach’s alpha value of the YMRS was 0.69.

^3^The Cronbach’s alpha value of the Positive and Negative Affect Schedule was 0.82.

^4^The Cronbach’s alpha value of the Brief QoL.BD was 0.87.

### Test of hypotheses

The Bayes factor results favored the null hypothesis, indicating no strong evidence for differences in creative achievement (CAQ) and the three psychological measures between participants with and without BD (see **Table 2**). In our sample, the mean scores on the Effort Discounting Task revealed that participants exhibited moderate sensitivity to reward magnitude but relatively low sensitivity to effort cost.

**Table 2:** Descriptive statistics and Bayes factors of creativity, ambition, and decision-making strategies.

	Bipolar (n = 44)		Non-bipolar (n = 69)		^1^BF_10_
	Mn	SD	Mn	SD	
^2^Log-transformed creativity (CAQ total)	3.77	1.33	4.10	1.24	0.45
Log-transformed painting creativity (CAQ)	2.18	1.78	2.62	1.68	0.44
Log-transformed writing creativity (CAQ)	1.17	1.53	1.62	1.49	0.60
Do people describe you as creative? (CAQ)	Yes, 23 (52.3%)		Yes, 30 (43.5%)		0.36
^3^Ambition (WASSUP)	15.67	3.98	15.29	4.33	0.23
Reward magnitude (Effort Discounting Task)	5.19	7.84	5.21	8.36	0.20
Effort cost (Effort Discounting Task)	-0.01	0.09	-0.03	0.11	0.25
^4^Explore-vs-exploit (Observe or Bet Task)	0.61	0.13	0.64	0.12	0.36

CAQ, Carson Creative Achievement Questionnaire; WASSUP, Willingly Approached Set of Statistically Unlikely Pursuits; Mn, mean; SD, standard deviation.

^1^Bayes factor of independent multinomial.

Null: The row and the column cell counts are independent; alternate: There is substantial dependency (relationship).

< 1: favors the null hypothesis; > 1: favors the alternate hypothesis; = 1: neutral.

^2^The Cronbach’s alpha value of the CAQ ranged from acceptable to good, with Cronbach’s alphas of α = 0.66 (writing) and α = 0.80 (painting), respectively.

^3^The Cronbach’s alpha value of the WASSUP scale was 0.66.

^4^The scores refer to “prop_good_bets” which reflects the proportion of bets an individual placed across different blocks and trials. Higher values indicate that the participant effectively balanced exploration and exploitation—exploring sufficiently to learn about the bias while also exploiting opportunities to maximize rewards.

**Figure 1:**
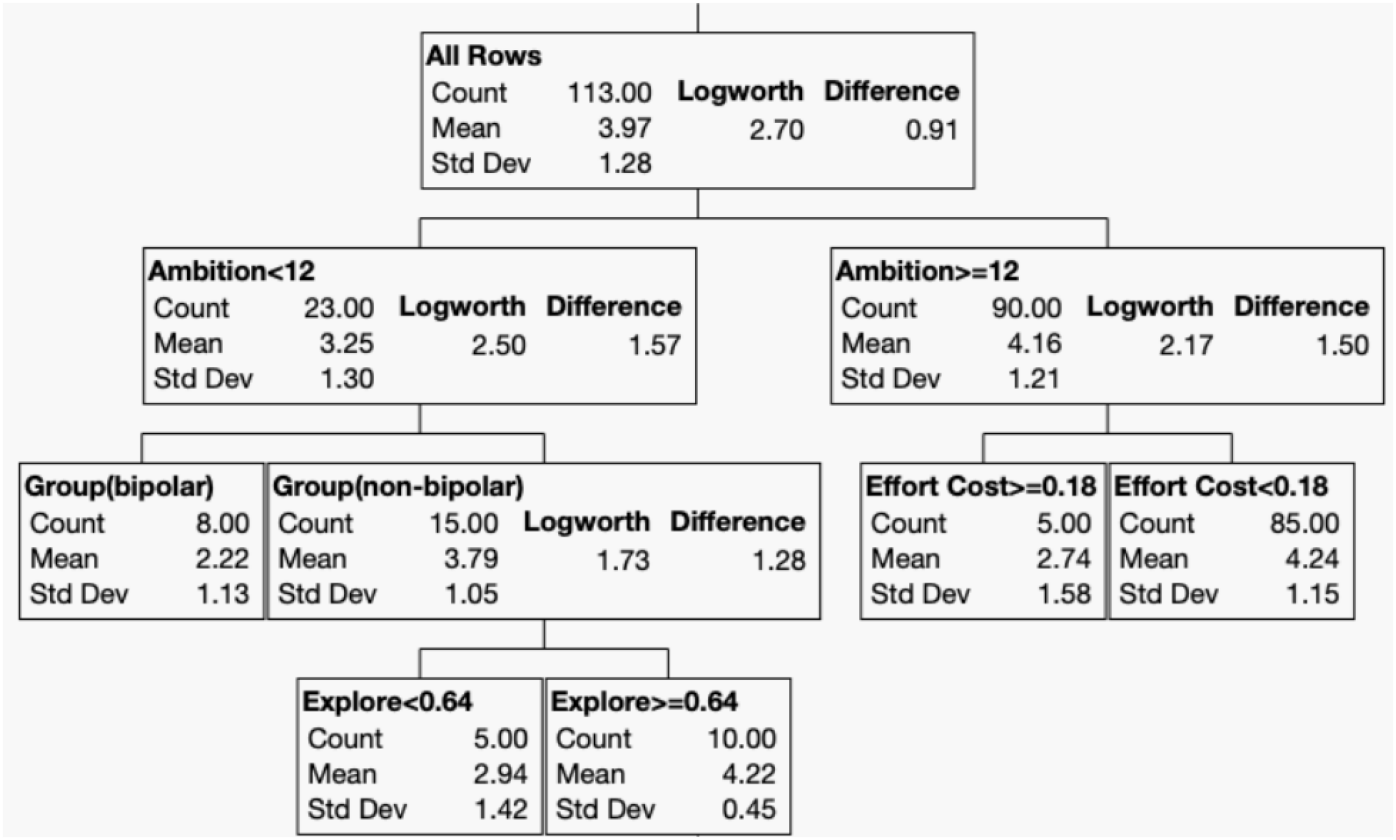
Decision tree algorithm using total creativity (CAQ creativity) as the dependent variable.

The decision-tree analysis, using log-transformed creativity scores (CAQ total) as the dependent variable, revealed several key interactions (**Figure 1**). The algorithm derived the cut-off and placed the greatest weight on ambition (cutoff = 12), with those scoring higher in regard to WASSUP showing greater creative accomplishment than those with lower WASSUP scores, regardless of BD status.

**Figure 2:**
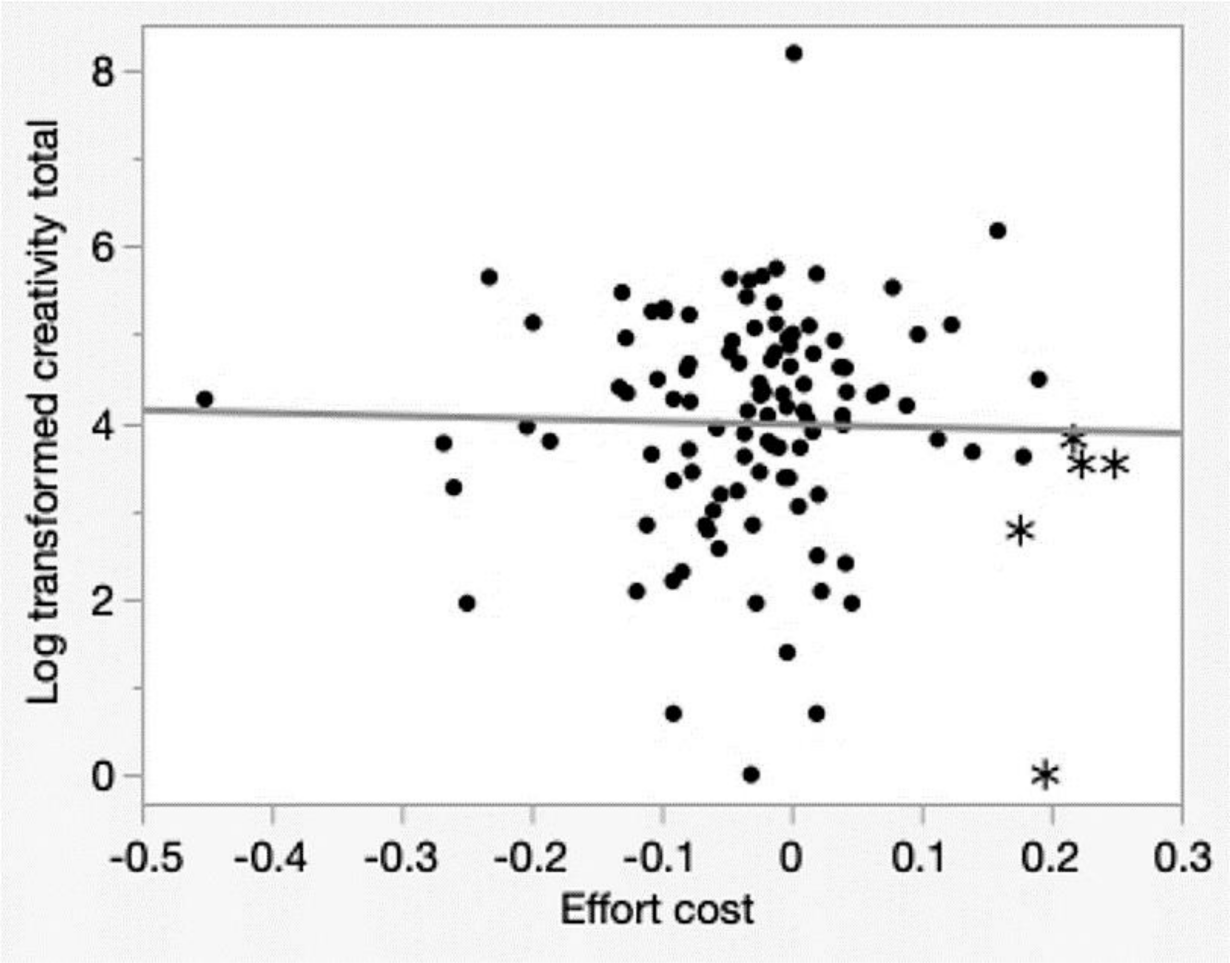
Scatter plot of total creativity and effort cost* with an ambition score >= 12*. Effort cost refers to the willingness to exert effort for a task. A "minus sign" effort cost indicates that individual readily applies force and tackles challenges, while a "non-minus sign" effort cost signifies that individual hesitates or delays before exerting effort. The round dots represent the 85 participants who were willing to persevere despite high physical demands; they achieved high creativity scores. In contrast, the star dots symbolize the five participants who devoted less effort; they had lower creativity scores.

Among participants with low ambition (< 12, n = 23), the non-BD group demonstrated higher creativity (total CAQ = 3.79, n = 15). For those without BD, a higher Explore versus Exploit score (indicating a better balance) correlated with greater creative achievement (total CAQ = 4.21, n = 10). However, for more ambitious participants (≥ 12, n = 90), diagnostic group membership (BD or non-BD) did not influence creativity. Instead, effort cost (cutoff = 0.18) emerged as a predictor; participants who persisted through challenging tasks, despite physical demands, achieved greater creativity (total CAQ = 4.24, n = 85). This is illustrated in the scatter plot in **Figure 2**.

Effort cost refers to the willingness to exert effort for a task.

## Discussion

The current study has several key strengths. It is among the first to examine the association between creativity and BD in an Asian population. It is also novel in conjointly considering several mechanisms that could help explain links between BD and creativity, using validated laboratory measures of decision-making. The work is strengthened by the inclusion of Bayesian analyses, decision-tree analyses, and data visualization, rather than over-relying on *p* values. Finally, the focus on writing and painting activities, objectively defined by time spent participating in these activities, is a strength compared to previous work, which has often relied on vague self-reported engagement in “creative work”.

In this study, we found no differences in creativity scores between participants with and without BD, supported by Bayesian comparisons and decision tree analyses. One possible explanation for the lack of differences in creativity levels is that individuals with BD in this sample were less likely to have completed postgraduate studies and were also less likely to be engaged in part-time or full-time employment compared to those without BD. These socioeconomic disadvantages, consistent with previous research (33, 34), may have created barriers that hindered the realization of creative potential. This suggests that the factors influencing creativity are more nuanced and complex than a simple relationship with educational attainment would imply.

Turning to the core questions of the present study, which were to understand mechanisms underlying creativity, we found that ambition, characterized by a strong desire to achieve success in difficult-to-attain life goals, was the most salient factor in predicting overall creativity scores. Research has highlighted the important role of ambition in determining creative outcomes, including among those with BD and those at high risk for BD (12, 13, 35). Ambition has been identified as a key variable in driving personal recovery from BD (36). The current work is novel in extending these findings to an Asian population.

The links between ambition and creative outcomes were modulated differently for those with high levels of ambition as compared to those with low levels of ambition. Notably, among participants with low ambition, BD status adversely affected their creativity outcomes. This could be because both low ambition and the presence of BD are tied to depressive symptoms, contributing to relatively low creative accomplishment in these subgroups. In contrast, among the highly ambitious participants, those who were willing to exert greater efforts for potential rewards in the effort-based decision-making task, despite increasing difficulty, demonstrated greater creative accomplishments. This is consistent with the notion that creative accomplishment requires “1% inspiration and 99% perspiration” (37).

Consistent with previous research, the findings of the two effort-based decision-making parameters showed separate effects. Although willingness to persevere in the face of high physical demands mattered, a willingness to work harder to earn more money was not related to creativity. Research has found that intrinsic motivation, rather than extrinsic motivation, is particularly important for creativity (38). Insensitivity to the level of monetary reward in this task has been linked to creativity task persistence in prior work (13). Our study suggests that working hard toward ambitious goals, rather than merely working hard for monetary gain, was the critical factor, which aligns with the inherent difficulty of attaining creative success, as well as the often-modest financial returns (at least in the initial stages) for most creative endeavors.

The influence of effort in regard to creativity was further modulated by explore versus exploit tendencies among the less ambitious group without BD. Those who showed greater investment in exploration (watchful observations in order to learn more) compared to exploitation (betting to earn in uncertain contexts) achieved more favorable creativity outcomes. This fits with recent theory (39) on the importance of the willingness to explore and seek novelty as a key part of the creative process.

These findings underscore the multifaceted nature of the relationships among ambition, decision-making, and creativity in individuals with BD as well as those without BD. A key implication is that, for those in recovery from BD seeking creative success, ambition may be a more important driver than diagnostic status. On the other hand, for less ambitious individuals who are not affected by BD, maintaining a balance between reward-seeking and exploration in decision-making can further support creative accomplishments.

Despite the novel findings, this study has several limitations. The relatively small sample size is of importance as it could contribute to the null diagnostic group effects. We acknowledge the imbalanced gender ratio, with fewer male participants, and that we observed a significant skew in creativity and effort levels, as few individuals exhibited extremely high, publicly recognized creative achievements. Creativity is ideally assessed by verifying public works and awards in addition to self-reported data. Additionally, some measures have only shown an acceptable level of internal consistency. To enhance the study of creativity and effort, future research should recruit larger, more representative community samples and focus on real-life decision-making strategies, such as how individuals navigate challenges, balance rewards and risks, and explore new opportunities.

In summary, ambition was found to be the most critical factor in creative outcomes, exceeding the impact of BD. These findings emphasize the critical balance required to support creativity—possessing ambitious goals, while demonstrating persistence when facing challenging tasks, and striking a balance between exploration and exploitation. To the best of our knowledge, this represents the first study to investigate these issues among a Chinese sample with and without BD.

### Supplementary information on measuring instruments

#### Carson creative achievement questionnaire

Our primary dependent variable was the CAQ, a self-report measure designed to assess lifetime creative accomplishments across 10 domains: visual arts, music, creative writing, dance, drama, architecture, humor, science, invention, and culinary skills (20). For each domain, participants recorded their level of accomplishment from zero (none) to seven. Total scores are based on the sum of the highest attainment in each category. We also calculated specific scores for accomplishments in the painting and writing domains (40). The CAQ scores correlate significantly with laboratory indices of creativity (40). We verified self-ratings through interviews. The original scale showed good convergent validity with divergent thinking tests (r = 0.47, p < 0.0001) and good test-retest reliability (r = 0.81, p < 0.0001) and internal consistency (= 0.96). The internal consistency reliability of the CAQ in the present study ranged from acceptable to good, with Cronbach’s alphas of α = 0.66 (writing) and α = 0.80 (painting), respectively.

The CAQ was augmented with a further item “Do people describe you as creative?” and were asked to respond either “yes” or “no” which was reported separately.

#### Willingly approached set of statistically unlikely pursuits: popular fame and financial success subscales

The WASSUP Popular Fame and Financial Success Subscales are designed to assess extremely high life ambitions (21). WASSUP scores have been found to be consistently elevated among people with BD (11) and those at risk of BD (35, 41), and to predict the onset of BD (42). Participants indicated how likely they were to set each of 10 highly ambitious life goals (e.g., “you will make HK$20 million or more”). The two subscales were summed to provide a total WASSUP score. Correlations with mood-related measures are as follows: popular fame (0.44, p < 0.001 for lifetime hypomania; -0.06 for lifetime depression; 0.21, p < 0.001 for current manic symptoms; -0.03 for current depression symptoms) and financial success (0.23, p < 0.001 for lifetime hypomania; -0.14, p < 0.001 for lifetime depression; 0.14, p < 0.001 for current manic symptoms; -0.08 for current depression symptoms). The internal consistency alpha coefficients were 0.85 for Popular Fame and 0.71 for Financial Success. The two scales were highly intercorrelated (r = 0.66). The Cronbach’s alpha value for the WASSUP scale in the present study was 0.66.

#### Effort discounting task

The Effort Discounting Task is a well-validated procedure designed to assess a person’s willingness to work harder to earn monetary rewards (17). The task was programmed in Python, with keyboard control, and a Biopac hand dynamometer TSD121c device with a DA100C transducer synchronized with the task delivery computer through the parallel port, using a MP160WSW data. Before the task began, participants were asked to squeeze the hand dynamometer as hard as they could for two seconds. Then, they were offered a chance to earn a bonus monetary reward if they could increase their grip pressure in a two-second round. This calibration was used to then index the level of effort, as a percentage of the maximum grip strength.

For each trial, participants were asked to choose between an easy-to-obtain small reward and a larger reward that required expending physical effort. In each block of 128 trials, participants chose between a “no grip” option and a larger reward that required squeezing the device at a given percentage of their maximum for two seconds. Trials varied in the degree of reward that could be earned (HK$0.5, $1, $2.5, or $5) and in the level of effort required (20%, 30%, 40%, 60%, or 80% of the individual’s maximum grip strength, as indexed with the hand dynamometer grip device) to earn the reward, so that individual regression parameters to quantify the sensitivity to the magnitude of reward, and to the effort required, could be extracted.

The monetary rewards accumulated as the task progressed were paid as a bonus at completion. Participants were remunerated up to HK$200 each for their performance in the task.

#### Observe or bet task

This task is designed to assess people’s tendency to make exploratory versus exploitative choices. In each block of 50 trials, the participants viewed a sequence of blue or orange lights, with one color biased to appear 75% of the time (18). In each trial, the participant made a choice between simply observing the light appear (an exploratory decision that did not yield any points) or guessing the color (“bet”, an exploit decision). During trials in which the participant chose to explore, they received feedback on the light color immediately. During trials in which the participant chose to exploit, the actual light color was not revealed immediately. Hence, exploratory decisions provided knowledge, but exploitative decisions had the potential to win or lose points. Thus, these “bet” decisions were risky and relied on the participant correctly deducing the bias. At a pseudo-random point in each block (between trials 15 and 35), there was a “change point”, where the bias switched. Participants took part in five blocks in total. At the end of each block, they received detailed feedback about their choices and the actual sequence of lights for each trial they had just completed, and then a “block summary” that included total correct bets, incorrect bets, observations, block winnings, and cumulative winnings.

#### Young mania rating scale

The YMRS is an interview-based rating scale designed to evaluate current manic symptom severity (22). The scale includes 11 items, most of which are scored on a scale ranging from zero to four (or zero to eight for items five to six and eight to nine), with higher numbers indicating greater symptom severity. A Trained researcher rated each item on the basis of participant responses and direct observations. This scale has been applied to Chinese clinical populations in previous studies (43). The Cronbach’s alpha value in the present study was 0.69.

#### Positive and negative affect schedule

The PANAS is a self-report questionnaire with 10 positive and 10 negative affect descriptors regarding mood and mental distress during the past week (23). Each item is rated on a five-point scale ranging from one (very slightly or not at all) to five (very much). Low PA scores indicate sadness and lethargy, while high PA scores indicate energy, concentration, and enjoyable engagement. Low NA scores represent calmness and serenity, while high NA scores reflect distress (44). The measure can be utilized within clinical and non-clinical populations (45). This scale has demonstrated good validity and reliability in studies with Chinese populations (46). The Cronbach’s alpha value for the PANAS in the current study was 0.82.

#### Brief version of quality of life in BD

The Brief QoL.BD is a well-validated self-report scale designed to capture QoL in individuals with BD across 14 domains (24, 25). It was iteratively developed with substantial consumer engagement (24). There is a Chinese version of the Brief QoL.BD, which has been validated and shows high internal consistency (Cronbach’s alpha = 0.82) and retest reliability (intraclass correlation coefficient = 0.808; 25). The Cronbach’s alpha value for the Brief QoL.BD in the present study was 0.87.

## Data Availability

The raw data supporting the conclusions of this article will be made available by the authors, without undue reservation.
